# Eupomatenoid-5 Isolated from Leaves of *Piper regnellii* Induces Apoptosis in *Leishmania amazonensis*

**DOI:** 10.1155/2013/940531

**Published:** 2013-03-21

**Authors:** Francielle Pelegrin Garcia, Danielle Lazarin-Bidóia, Tânia Ueda-Nakamura, Sueli de Oliveira Silva, Celso Vataru Nakamura

**Affiliations:** ^1^Programa de Pós Graduação em Ciências Biológicas, Laboratório de Inovação Tecnológica no Desenvolvimento de Fármacos e Cosméticos, Bloco B-08, Universidade Estadual de Maringá, Avenida Colombo 5790, 87020-900 Maringá, PR, Brazil; ^2^Programa de Pós Graduação em Ciências Farmacêuticas, Universidade Estadual de Maringá, Avenida Colombo 5790, 87020-900 Maringá, PR, Brazil

## Abstract

*Leishmania* spp. are protozoa responsible for leishmaniasis, a neglected disease that kills up to 50,000 people every year. Current therapies mainly rely on antimonial drugs that are inadequate because of their poor efficacy and safety and increased drug resistance. An urgent need exists to find new and more affordable drugs. Our previous study demonstrated the antileishmanial activity of eupomatenoid-5, a neolignan obtained from leaves of *Piper regnellii* var. *pallescens*. The aim of the present study was to clarify the mode of action of eupomatenoid-5 against *L. amazonensis*. We used biochemical and morphological techniques and demonstrated that eupomatenoid-5 induced cell death in *L. amazonensis* promastigotes, sharing some phenotypic features observed in metazoan apoptosis, including increased reactive oxygen species production, hypopolarization of mitochondrial potential, phosphatidylserine exposure, decreased cell volume, and G0/G1 phase cell cycle arrest.

## 1. Introduction

Leishmaniasis remains a significant neglected tropical disease that puts 350 million people in 88 countries on four continents at risk for infection, with approximately 50,000 deaths per year [1]. Approximately 21 species have been found to cause three different clinical manifestations of leishmaniasis: cutaneous, in which the lesions are confined to the site of inoculation by the sandfly, and mucocutaneous, which affects mucosal tissues, and visceral leishmaniasis affecting the inner organs [2].

Current chemotherapy for leishmaniasis is still based on the use of pentavalent antimonials as first-line drugs and pentamidine, amphotericin B (free or liposomal forms), paromomycin, and miltefosine as second-line drugs. Although these drugs are usually effective, they have limitations, such as toxicity in the host and long-term treatment [3, 4]. These drawbacks reveal the urgent need to develop new therapeutic agents for the treatment of leishmaniasis. 

Natural compounds, known to be valuable sources of new medicinal agents, have been exhaustively evaluated against *Leishmania* [5–7]. One example is eupomatenoid-5, a neolignan obtained from the leaves of *Piper regnellii* var. *pallescens,* representing a subclass with various biological activities, such as antifungal, antibacterial, insecticidal, and trypanocidal effects [8–12]. Our previous study reported the antileishmanial effect of eupomatenoid-5 [13]. Considering such antileishmanial activity, the aim of the present study was to better characterize the biochemical alterations induced by this compound against promastigote forms of *L. amazonensis *and elucidate the mechanism of action of eupomatenoid-5 involved in the cell death of this protozoan parasite.

## 2. Materials and Methods

### 2.1. Chemicals

Actinomycin D, antimycin A, bovine serum albumin, carbonyl cyanide m-chlorophenylhydrazone (CCCP), digitonin, dimethylsulfoxide (DMSO), rhodamine 123 (Rh123), 2′,7′-dichlorofluorescin diacetate (H_2_DCFDA), and 5-5′-dithio*bis*(2-nitrobenzoic acid) (DTNB) were purchased from Sigma-Aldrich (St. Louis, MO, USA). Fetal bovine serum (FBS) was obtained from Invitrogen (Grand Island, NY, USA). Annexin-V FITC, 3,8-phenanthridine diamine-5-(6-triphenylphosphonium hexyl)-5,6-dihydro-6-phenyl (MitoSOX) and propidium iodide (PI) were obtained from Invitrogen (Eugene, OR, USA). The protein assay kit was obtained from Bio-Rad (Hercules, CA, USA). All of the other reagents were of analytical grade.

### 2.2. Isolation of Eupomatenoid-5 from Leaves of *Piper regnellii* Var. *pallescens*

**Figure 1:**
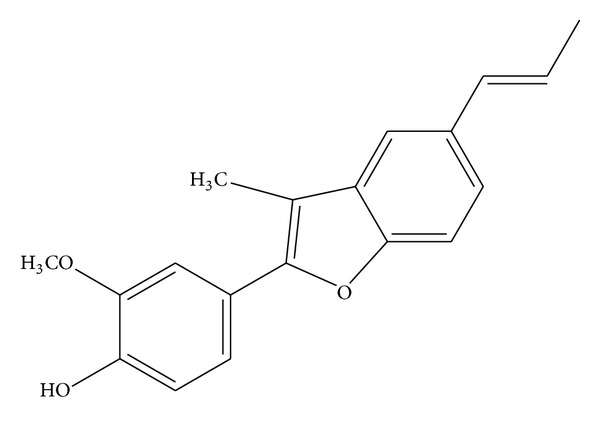
Structure of eupomatenoid-5 isolated from the leaves of *Piper regnellii *var. *pallescens*.

Eupomatenoid-5 (Figure 1) was isolated from the leaves of *P. regnellii*, which was collected in the Professor Irenice Silva Garden of Medicinal Plants on the campus of the State University of Maringa (UEM) in Parana, Brazil. A voucher specimen (no. HUM 8392) was deposited at the UEM Herbarium. Briefly, the dry plant material was extracted by exhaustive maceration at room temperature in the dark in an ethanol : water ratio of 90 : 10. Fractionation was performed from the ethyl acetate extract to obtain the hexane fraction, and a dihydrobenzofuran neolignan, eupomatenoid-5, was isolated from this fraction as described previously [9]. The compound was purified using absorption-chromatographic methods and identified by analyzing the ultraviolet, infrared, ^1^H nuclear magnetic resonance (NMR), ^13^C NMR, distortionless enhancement polarization transfer (DEPT), correlated spectroscopy (COSY), heteronuclear correlation (HETCOR), nuclear overhauser effect spectroscopy (NOESY), heteronuclear multiple bond correlation (HMBC), and gas chromatography/mass spectrometry (GC/MS) spectra. The data were compared with the literature [14].

Stock solutions of eupomatenoid-5 were prepared aseptically in DMSO and diluted in culture medium so that the DMSO concentration did not exceed 1% in the experiments. The concentrations of eupomatenoid-5 used in the assays were 30.0, 85.0, and 170.0 *μ*M, representing the IC_50_, IC_90_, and twofold IC_90_, respectively [13].

### 2.3. Parasites

*Leishmania amazonensis* promastigotes (MHOM/BR/Josefa) were maintained at 25°C in Warren's medium (brain-heart infusion plus hemin and folic acid; pH 7.2) supplemented with 10% heat-inactivated FBS.

### 2.4. Determination of Mitochondrial Transmembrane Potential (ΔΨ*m*)

Promastigotes (1 × 10^7^ cells/mL) were treated or untreated with 30.0, 85.0, and 170.0 *μ*M eupomatenoid-5 for 24 h at 37°C, harvested, and washed with phosphate-buffered saline (PBS). The parasites were incubated with 1 mL (5 mg/mL in ethanol) of Rh123, a fluorescent probe that accumulates in mitochondria, for 15 min, resuspended in 0.5 mL PBS, and incubated for an additional 30 min. The assay was conducted according to the manufacturer's instructions. The parasites were analyzed using a BD FACSCalibur flow cytometer and CellQuest Pro software (Becton Dickinson and Company, USA, 1997). A total of 10,000 events were acquired in the region that corresponded to the parasites. CCCP at 100 *μ*M was used as a positive control [15].

### 2.5. Measurement of Reactive Oxygen Species

Promastigotes (1 × 10^7^ cells/mL) were treated or untreated with 30.0, 85.0, and 170.0 *μ*M for 3 and 24 h, centrifuged, washed, and resuspended in PBS (pH 7.4). Afterward, these parasites were loaded with 10 *μ*M of a permeant probe, H_2_DCFDA, in the dark for 45 min [16]. Reactive oxygen species (ROS) were measured as an increase in fluorescence caused by the conversion of nonfluorescent dye to highly florescent 20,70-dichlorofluorescein, with an excitation wavelength of 488 nm and emission wavelength of 530 nm in a fluorescence microplate reader (Victor X3, PerkinElmer, Finland).

### 2.6. Fluorimetric Detection of Mitochondrial-Derived O_2_^•−^

Promastigotes (2 × 10^7^ cells/mL) were loaded with 5 *μ*M of a fluorescent O_2_^•−^-sensitive, mitochondrial-targeted probe, MitoSOX, for 10 min at 25°C and then washed with Krebs-Henseleit (KH) buffer (pH 7.3) that contained 15 mM NaHCO_3_, 5 mM KCl, 120 mM NaCl, 0.7 mM Na_2_HPO_4_, and 1.5 mM NaH_2_PO_4_ [17]. After the parasites were treated or untreated with 30.0, 85.0, and 170.0 *μ*M eupomatenoid-5, the fluorescence intensity was detected after 1, 2, 3, and 4 h of treatment using a fluorescence microplate reader (Victor X3, PerkinElmer, Finland), with an excitation wavelength of 510 nm and emission wavelength of 580 nm. Oxidized MitoSOX (oxMitoSOX) becomes highly fluorescent upon binding to nucleic acids. Cells were exposed to 10 *μ*M antimycin A, a stimulus known to induce mitochondrial O_2_^•−^ production.

### 2.7. Estimation of Decrease in Reduced Thiol Level

Thiol levels were determined using DTNB. Promastigotes (1 × 10^7^ cells/mL) were treated or untreated with 30.0, 85.0, and 170.0 *μ*M eupomatenoid-5 for 24, 48, and 72 h at 25°C. Afterward, the parasites were centrifuged, dissolved in 10 mM Tris-HCl buffer (pH 2.5), and sonicated. Acidic pH was used during sonication to prevent oxidation of the free thiol groups. Cellular debris was removed by centrifugation, and 100 *μ*L of the supernatant and 100 *μ*L of 500 mM phosphate buffer (pH 7.5) were taken in each microtiter well, followed by the addition of 20 *μ*L of 1 mM DTNB to each well. Absorbance was measured at 412 nm [16].

### 2.8. Phosphatidylserine Exposure

Promastigotes (1 × 10^7^ cells/mL) were treated or untreated with 30.0, 85.0, and 170.0 *μ*M eupomatenoid-5 for 24 h at 25°C. Afterward, the parasites were washed and resuspended in 100 *µ*L of binding buffer (140 mM NaCl, 5 mM CaCl_2_, and 10 mM HEPES-Na, pH 7.4), followed by the addition of 5 *μ*L of a calcium-dependent phospholipid binding protein, annexin-V FITC, for 15 min at room temperature. Binding buffer (400 *μ*L) and 50 *μ*L PI were then added. Antimycin A (125.0 *μ*M) was used as a positive control. Data acquisition and analysis were performed using a BD FACSCalibur flow cytometer equipped with CellQuest software. A total of 10,000 events were acquired in the region that was previously established as the one that corresponded to the parasites. Cells that were stained with annexin-V (PI-positive or -negative) were considered apoptotic, and cells that were only PI-positive were considered necrotic [18].

### 2.9. Determination of Cell Volume of Parasites

Promastigotes (1 × 10^7^ cells/mL) were treated or untreated with 30.0, 85.0, and 170.0 *μ*M eupomatenoid-5 for 24 h at 25°C, harvested, and washed with PBS. Subsequently, the parasites were analyzed using a BD FACSCalibur flow cytometer and CellQuest Pro software. Histograms were generated, and FSC-H represented the cell volume. A total of 10,000 events were acquired in the region that corresponded to the parasites. Actinomycin D (20.0 mM) was used as a positive control [19].

### 2.10. Determination of Cellular Membrane Integrity

Promastigotes (1 × 10^7^ cells/mL) were treated or untreated with 30.0, 85.0, and 170.0 *μ*M eupomatenoid-5 for 24 h at 32°C, harvested, and washed with PBS. The parasites were incubated with 50 mL of 2 mg/mL PI for 5 min according to the instructions provided by the manufacturer. Immediately thereafter, the parasites were analyzed using a BD FACSCalibur flow cytometer equipped with CellQuest software. A total of 10,000 events were acquired in the region that corresponded to the parasites. Digitonin (40.0 *μ*M) was used as a positive control [19].

### 2.11. Scanning Electron Microscopy

Promastigotes (1 × 10^6^ cells/mL) were treated or untreated with 30.0 and 85.0 *μ*M eupomatenoid-5 for 48 h at 25°C and fixed in 2.5% glutaraldehyde in 0.1 M sodium cacodylate buffer for 1–3 h. Subsequently, the parasites were adhered on poly-L-lysine-coated coverslips and dehydrated in increasing concentrations of ethanol. The samples were critical point dried in CO_2_, coated with gold, and observed in a Shimadzu SS-550 (Japan) scanning electron microscope [20].

### 2.12. Cell Cycle

Promastigotes (1 × 10^7^ cells/mL) were treated or untreated with 30.0, 85.0, and 170.0 *μ*M eupomatenoid-5 for 24 h at 25°C. After incubation, the parasites were centrifuged and washed twice in PBS (pH 7.4). The resultant pellet was resuspended in 500 *μ*L of a cold methanol/PBS (70% v/v) mixture and maintained at 4°C for 1 h. Afterward, the pellet was centrifuged, resuspended in PBS with 10 *μ*g/mL PI and 20 *μ*g/mL of DNase-free RNase (200 mg), and incubated for 45 min at 37°C. Data were acquired using a BD FACSCalibur flow cytometer and analyzed using CellQuest Pro software [21].

### 2.13. Statistical Analysis

The data shown in the graphs are expressed as the means ± standard deviation (SD) of the mean of at least three independent experiments. The data were analyzed using one- and two-way analysis of variance (ANOVA). Significant differences among means were identified using the Tukey *post hoc* test. Values of *P* ≤ 0.05 were considered statistically significant. Statistical analyses were performed using Prism 5 (GraphPad Software, San Diego, CA, USA, 2007).

## 3. Results

### 3.1. Mitochondrial Membrane Potential

**Figure 2:**
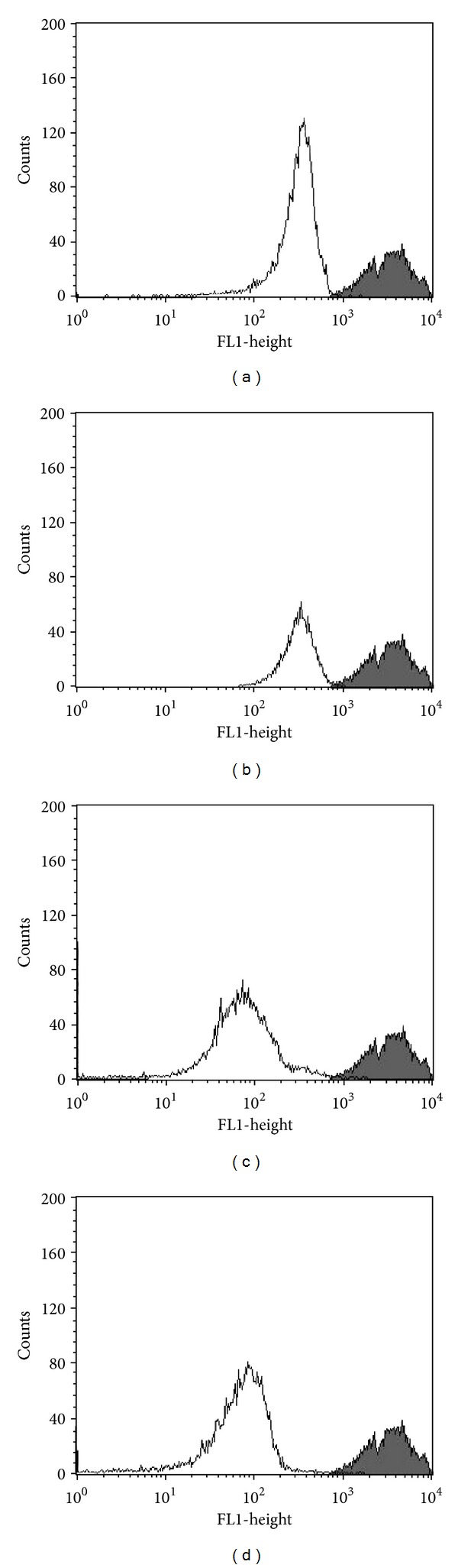
Mitochondrial membrane potential assay in promastigote forms of *L. amazonensis *treated with eupomatenoid-5 at (b) 30.0 *μ*M, (c) 85.0 *μ*M, and (d) 170.0 *μ*M for 24 h and stained with Rh123, which accumulates in mitochondria. (a) Positive control (CCCP). The gray area corresponds to the control group (i.e., untreated parasites). Typical histograms of at least three independent experiments are shown.

Our previous study used transmission electron microscopy and found that eupomatenoid-5 caused damage and significant changes in promastigote mitochondria [13]. Based on this, we decided to evaluate the ΔΨm in eupomatenoid-5-treated parasites using flow cytometry and Rh123, a fluorescent marker that indicates mitochondrial membrane potential. The histograms showed that eupomatenoid-5 decreased total Rh123 fluorescence intensity at all of the concentrations tested compared with the control group, indicating mitochondrial depolarization (Figures 2(a)–2(c)). This loss of ΔΨm was higher at the IC_90_ (95.9%) than that at the IC_50_ (76.8%), and at the twofold IC_90_, the loss of ΔΨm remained the same as the IC_90_ (94.9%). The positive control, CCCP, induced a decrease of 62.5% in mitochondrial membrane potential.

### 3.2. Mitochondrial-Derived O_2_^•−^ Production

**Figure 3:**
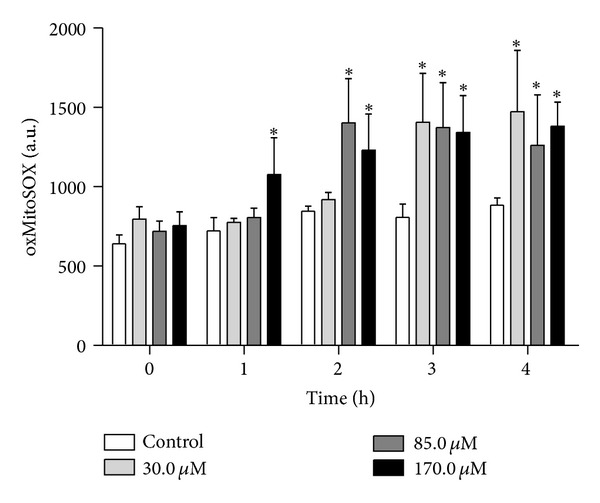
Mitochondrial O_2_^•−^ production in promastigote forms of* L. amazonensis *treated with eupomatenoid-5 at 30.0, 85.0, and 170.0 *μ*M for up to 4 h using the fluorescence probe MitoSOX. At the indicated times, parasites were used to fluorometrically measure oxidized MitoSOX (oxMitoSOX). The data are expressed as the mean fluorescence (in arbitrary units) ± SD of at least three independent experiments. *P ≤ 0.05, significant difference compared with the control group (i.e., untreated parasites).

Based on our ΔΨm results, we evaluated O_2_^•−^ production in eupomatenoid-5-treated parasites. Mitochondrial-derived O_2_^•−^ production was evaluated using MitoSOX reagent, which measures the mitochondrial accumulation of superoxide based on its hydrophobic nature and positively charged triphenylphosphonium moiety [20].  Figure 3 shows that eupomatenoid-5 significantly increased the production of mitochondrial O_2_^•−^ at most of the concentrations and times tested compared with the control group. After 1 and 2 h of treatment, eupomatenoid-5 increased O_2_^•−^ production by approximately 40% only at higher concentrations. In contrast, after 3 and 4 h of treatment, eupomatenoid-5 increased O_2_^•−^ production by more than 50% at all of the concentrations tested. The positive control, antimycin A, induced a two-fold increase in mitochondrial O_2_^•−^ production.

### 3.3. Reactive Oxygen Species Level

**Figure 4:**
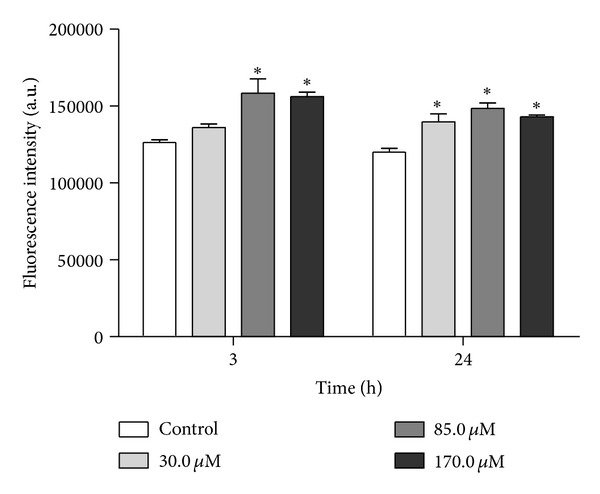
Total ROS production in promastigote forms of* L. amazonensis *treated with eupomatenoid-5 at 30.0, 85.0, and 170.0 *μ*M for 3 and 24 h using the fluorescence probe H_2_DCFDA. The data are expressed as the mean fluorescence (in arbitrary units) ± SD of at least three independent experiments. *P ≤ 0.05, significant difference compared with the control group (i.e., untreated parasites).

Based on the MitoSOX data, we evaluated the effects of total ROS production in eupomatenoid-5-treated parasites using a fluorescent probe, H_2_DCFDA. This probe primarily detects H_2_O_2_ and hydroxyl radicals and fluoresces after forming dichlorofluorescein [22]. Our results showed that eupomatenoid-5 increased total ROS production at all of the concentrations and times tested compared with the control group (Figure 4). However, a significant increase in total ROS production of approximately 25% was observed after 3 h of treatment at 85.0 and 170.0 *μ*M. After 24 h treatment, total ROS production increased by 16.3% even at the lower concentration.

### 3.4. Reduced Thiol Levels

**Figure 5:**
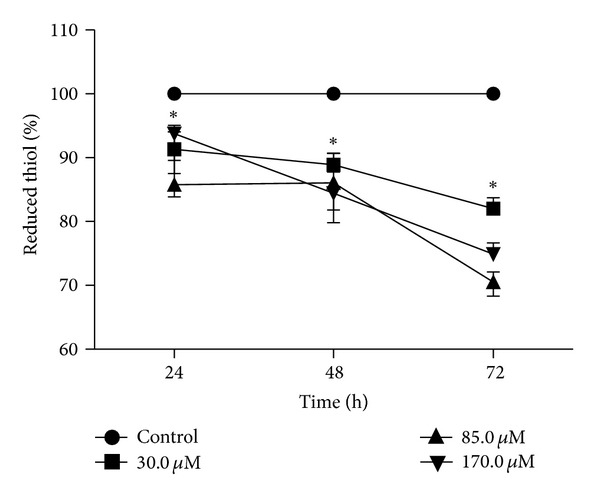
Thiol levels in promastigote forms of *L. amazonensis *treated with eupomatenoid-5 at 30.0, 85.0, and 170.0 *μ*M for 24, 48, and 72 h using DTNB. The data are expressed as the mean ± SD of at least three independent experiments. *P ≤ 0.05, significant difference compared with the control group (i.e., untreated parasites).

Our data suggest that eupomatenoid-5 induces oxidative imbalance, attributable to enhanced ROS production. However, oxidative imbalance conditions depend on both increased oxidant species and decreased antioxidant effectiveness [23]. Therefore our next step was to assess the effect of eupomatenoid-5 on the level of reduced thiols, which might be decreased, for example, by a reduction in trypanothione reductase (TR) activity. Eupomatenoid-5 dose-dependently decreased total reduced thiol levels at all of the concentrations and times tested compared with the control group (Figure 5). This decrease might also be considered time-dependent, with reductions of thiol levels of approximately 10, 15, and 20% after 24, 48, and 72 h treatment, respectively.

### 3.5. Phosphatidylserine Exposure

**Figure 6:**
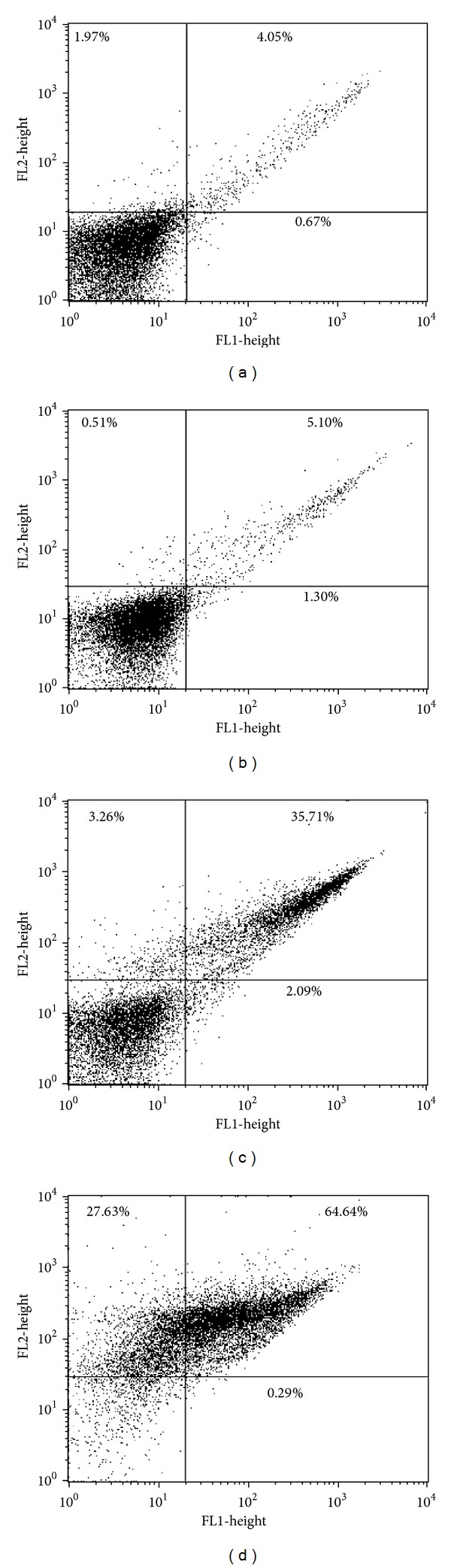
Phosphatidylserine exposure in promastigote forms of* L. amazonensis* treated with eupomatenoid-5 at (b) 30.0 *μ*M, (c) 85.0 *μ*M, and (d) 170.0 *μ*M for 24 h using annexin-V FITC and PI. (a) corresponds to the control group (i.e., untreated parasites). The results are presented in a dot plot together with the negative control. Typical histograms of at least three independent experiments are shown.

To determine whether the mechanism of cell death triggered by eupomatenoid-5 involves apoptosis, we evaluated the externalization of phosphatidylserine, an apoptotic marker that is present in the outer leaflet of plasmalemma [24] in promastigotes treated with eupomatenoid-5 for 24 h and double stained with FITC-conjugated annexin-V and PI. As shown in Figure 6, eupomatenoid-5 increased annexin-V fluorescence intensity more than 30% at higher concentrations (85.0 and 170.0 *μ*M) compared with the control group, indicating phosphatidylserine exposure (Figures 6(c) and 6(d)).

### 3.6. Cell Volume

**Figure 7:**
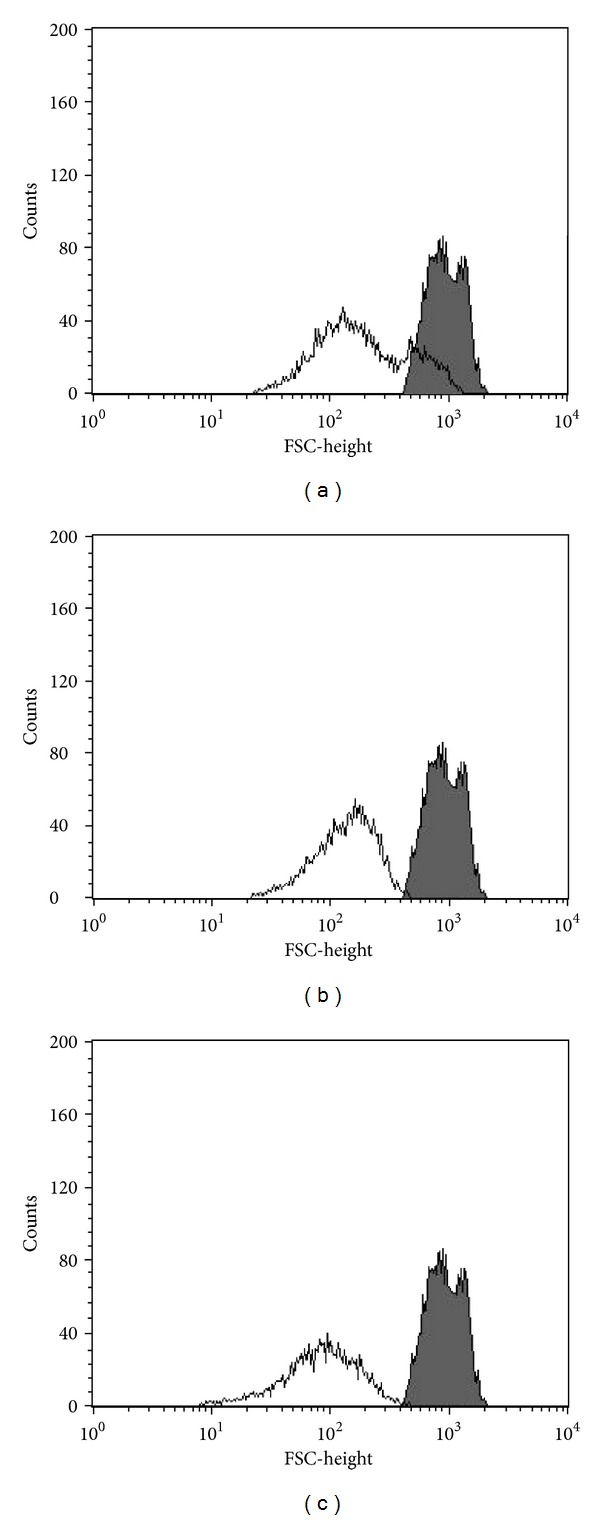
Cell volume in promastigote forms of *L. amazonensis *treated with eupomatenoid-5 at (a) 30.0 *μ*M, (b) 85.0 *μ*M, and (c) 170.0 *μ*M. FSC-H was considered a function of cell size. The gray area corresponds to the control group (i.e., untreated parasites). Typical histograms of at least three independent experiments are shown.

In addition to biochemical alterations, apoptosis also induces morphological alterations. Based on this, we evaluated cell shrinkage, a hallmark of apoptotic death, in eupomatenoid-5-treated parasites [25]. As shown in Figure 7, a dose-dependent decrease in cell volume (30.4, 63.1, and 84.3%, resp.) was observed at all of the concentrations tested compared with the control group.

### 3.7. Cell Membrane Integrity

**Figure 8:**
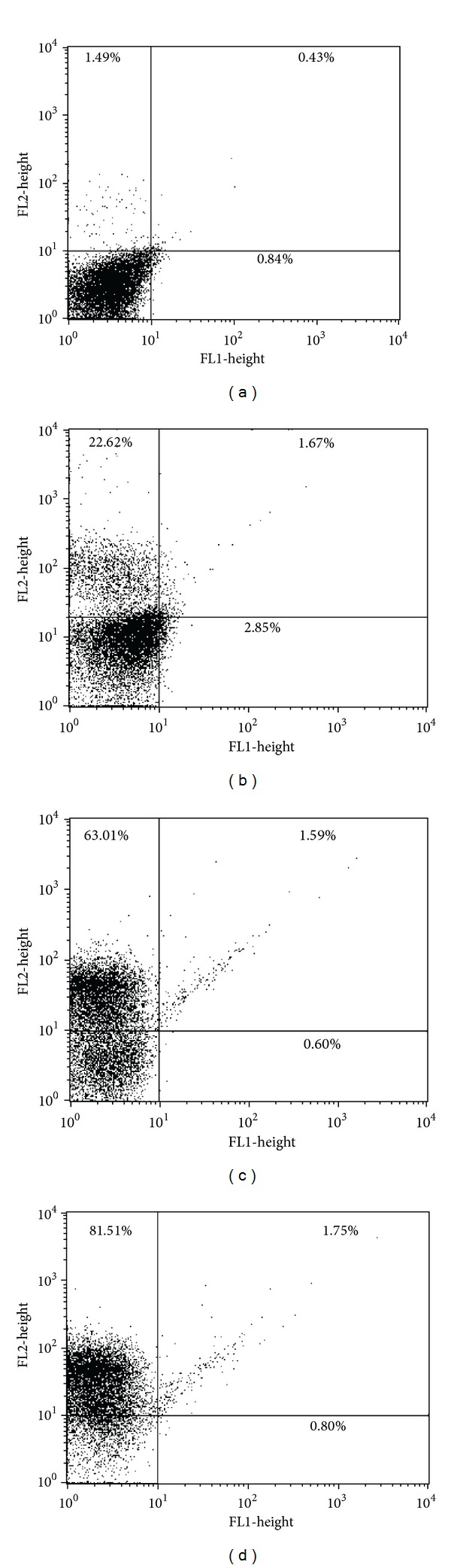
Cell membrane integrity assay in promastigote forms of *L. amazonensis *treated with eupomatenoid-5 at (b) 30.0 *μ*M, (c) 85.0 *μ*M, and (d) 170.0 *μ*M for 24 h and stained with PI. The numbers show the percentage of PI-positive parasites in (a) and (b). (a) corresponds to the control group (i.e., untreated parasites). Typical histograms of at least three independent experiments are shown.

To determine whether the mechanism of cell death triggered by eupomatenoid-5 also involves the necrotic death pathway, we evaluated plasma membrane integrity in eupomatenoid-5-treated promastigotes stained with PI, which diffuse across permeable membranes and bind to nucleic acids. As shown in Figure 8, eupomatenoid-5 at 30.0, 85.0, and 170.0 *μ*M dose-dependently increased PI-stained parasites from 1.9% (untreated promastigotes; Figure 8(a), upper quadrants) to 24.3%, 64.6%, and 86.3%, respectively (Figures 8(b)–8(d), upper quadrants), indicating permeabilization of the plasma membrane. The positive control, digitonin, showed a 47.7% increase in the gated percentage of PI-stained cells.

### 3.8. Scanning Electron Microscopy

**Figure 9:**
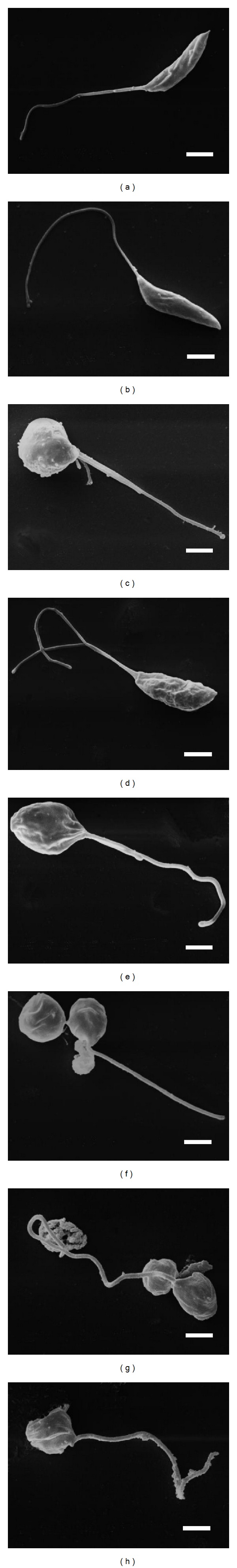
Scanning electron microscopy images of promastigote forms of *L. amazonensis *incubated in the ((a) and (b)) absence or presence of eupomatenoid-5 at ((c)–(e)) 30.0 *μ*M and ((f)–(h)) 85.0 *μ*M for 48 h. Scale bar = 2 *μ*m.

To confirm that eupomatenoid-5 induced morphological alterations in promastigotes, we further evaluated morphological alterations using scanning electron microscopy. Photomicrographs revealed that untreated protozoa had typical characteristics, with an elongated shape and terminal flagellum. In contrast, eupomatenoid-5 dose-dependently altered the size and shape of the treated parasites, including a reduction and rounding of the cellular body (Figure 9).

### 3.9. Cell Cycle

To evaluate the ratio of pseudohypodiploid cells, flow cytometry after cell permeabilization and PI labeling were used. In a given cell, the amount of bound dye correlates with the DNA content, and thus DNA fragmentation in apoptotic cells is reflected by fluorescence intensity that is lower than that of G0/G1 cells (i.e., a sub-G0/G1 peak) [26]. The incubation of promastigotes with eupomatenoid-5 for 3 and 24 h resulted in a 16% increase in the proportion of cells in the sub-G0/G1 phase at the lower concentration (30.0 *μ*M) and a 28% increase at the higher concentrations (85.0 and 170.0 *μ*M) compared with the control group (Table 1). In contrast, an increase in the number of cells in the sub-G0/G1 phase led to a decrease in the number of cells in the G2/M phase compared with untreated cells.

## 4. Discussion

Despite recent advances, the treatment of leishmaniasis continues to be unsatisfactory. Pentavalent antimonials remain the first-line treatment for this infection in most endemic areas, despite their limitations, such as high toxicity and increased drug resistance [27, 28]. Thus, an urgent need exists to develop new drugs and therapeutic strategies. We have been extensively exploring plant resources to find effective antileishmanial agents. Our previous studies indicated that eupomatenoid-5 contained in the crude extracts and chloroform fractions of *P. regnellii* leaves are responsible for the antileishmanial activity of this plant [13]. Furthermore, eupomatenoid-5 showed more selective against protozoan than macrophage cells [13]. Additionally, transmission electron microscopy revealed many ultrastructural alterations, especially in the mitochondria of treated parasites, indicating damage and significant changes in this organelle [13]. The present study sought to further elucidate the mechanism of action of eupomatenoid-5 in the cell death of this protozoan.

We initially focused on investigating mitochondrial alterations and their consequences, especially with regard to parasite death. We found that parasites treated with eupomatenoid-5 exhibited a decrease in ΔΨm and increase in mitochondrial ROS production. *Leishmania* is known to have a single mitochondrion, and so the maintenance of mitochondrial transmembrane potential is essential for parasite survival [29]. In fact, a number of studies have been published describing compounds that target at Trypanosomatids mitochondria [16, 30]. Eupomatenoid-5 also induced a time-dependent decrease in reduced thiol levels of treated parasites. The trypanothione system is unique in trypanosomatid parasites and plays an important role in the homeostasis of parasite redox metabolism [31, 32]. In this system, trypanothione is reduced to a dithiol T(SH)2 by trypanothione/trypanothione reductase (TR). The inhibition of TR decreases total reduced thiol [16]. Our data suggest that eupomatenoid-5 induces oxidative imbalance in promastigote forms through two pathways, increasing ROS production by disrupted mitochondria and decreasing hydroperoxide detoxification by reducing TR activity. 

Additionally, eupomatenoid-5 induced the externalization of phosphatidylserine and a reduction of parasite volume, indicated by flow cytometry and scanning electron microscopy. Altogether, our data showed that eupomatenoid-5-treated parasites exhibited apoptotic-like events. An increase in the generation of ROS in the cytosol, an established event in most apoptotic cells, might direct the cell toward this death pathway [33]. Interestingly, programmed cell death in protists appears to share some morphological features with apoptosis in multicellular organisms, including cell shrinkage, the loss of mitochondrial membrane potential, and the externalization of phosphatidylserine [34, 35]. Following phosphatidylserine flip, apoptotic cells lose their plasma membrane integrity. This signal was also induced by eupomatenoid-5 and revealed by the addition of PI, a cellimpermeable nuclear dye [36]. Interestingly, similar results were found in parasitic forms of *Trypanosoma cruzi *after treatment with eupomatenoid-5 [19].

Finally, we demonstrated that eupomatenoid-5 induced G0/G1 phase cell cycle arrest using flow cytometry and PI labeling, in which the amount of bound dye correlated with the DNA content, and thus DNA fragmentation in apoptotic cells is translated into fluorescence intensity that was lower than that of G0/G1 cells (i.e., a sub-G0/G1 peak) [26, 37].

## 5. Conclusion

In parasites, apoptosis appears to be the predominant form of cell death [38], which has been observed in kinetoplastids in response to chemotherapeutic agents, such as amphotericin B and plant extracts, such as Aloe Vera leaf exudates [39, 40]. We used biochemical and morphological techniques and demonstrated that eupomatenoid-5 induced cell death, sharing some phenotypic features observed in metazoan apoptosis, including increased ROS, hypopolarization of mitochondrial potential, phosphatidylserine exposure, a reduction of cell volume, and G0/G1 phase cell cycle arrest. The study of the major pathways involved in *Leishmania* apoptosis-like death will provide insights into the future design of newer chemotherapeutic strategies.

## Figures and Tables

**Table 1:** Effect of eupomatenoid-5 on the cell cycle of *L. amazonensis *promastigotes (1 × 10^7^  cells/mL) that were treated with 30.0, 85.0, and 170.0 *μ*M eupomatenoid-5 for 24 h and processed for cell cycle analysis as described in Section 2. The data are expressed as percentages.

Group	Sub-G0/G1 (M1)	G0/G1 (M2)	S and G2/M (M3)
Control	4.16	57.31	37.16
30.0 *μ*M	16.28	55.29	21.20
85.0 *μ*M	28.11*	47.03	16.96
170.0 *μ*M	28.41*	45.44	15.84
